# Prevalence and Risk Factors for Enlarged Perivascular Spaces in Young Adults from a Neurology Clinic-Based Cohort

**DOI:** 10.3390/brainsci12091164

**Published:** 2022-08-30

**Authors:** Qiaoqiao Zou, Mingliang Wang, Xiaoer Wei, Wenbin Li

**Affiliations:** Department of Radiology, Shanghai Jiao Tong University Affiliated Sixth People’s Hospital, Shanghai 200233, China

**Keywords:** enlarged perivascular space, young patients, symptoms, MRI, small vessel disease

## Abstract

(1) Background: This study aimed to investigate the prevalence and risk factors for enlarged perivascular spaces (EPVS) in young adults from a neurology clinic-based cohort (≤45 years old) via unenhanced brain MRI. (2) Methods: A total of 931 young adults from a neurology clinic-based cohort who underwent unenhanced brain MRI between 1 January 2021 and 30 June 2021 were retrospectively included in this study. The EPVS were rated in the centrum semiovale (CSO-EPVS), basal ganglia (BG-EPVS), and midbrain (MB-EPVS) using a visual rating scale. The degrees of the CSO-EPVS, BG-EPVS, and MB-EPVS were all divided by a cutoff value of 1. Demographic factors, vascular risk factors, and symptoms were analyzed using the chi-square test and logistic regression to determine the risk factors of EPVS. (3) Results: The overall prevalence of EPVS was 99.8% (929/931). The CSO-EPVS, BG-EPVS, and MB-EPVS were predominantly scored as 1 (52.1%, 79.1%, and 58.3%, respectively). Logistic regression analysis identified age and hypertension as factors affecting the degrees of CSO-EPVS and BG-EPVS (*p* < 0.05). Hypertension (*p* < 0.001) and diabetes (*p* = 0.014) were revealed to be factors affecting the degree of BG-EPVS. Furthermore, patients with headache (OR = 1.807; *p* = 0.001) and dizziness (OR = 1.574; *p* = 0.025) were associated with MB-EPVS. (4) Conclusions: EPVS were frequently found in young adults and could be related to the symptoms. Age, hypertension, and diabetes were the risk factors for the severity of EPVS in the corresponding brain regions.

## 1. Introduction

Currently, there are an increasing number of clinical applications for non-enhanced MRIs of the brain; enlarged perivascular spaces (EPVS) are frequently observed in clinical patients with cerebral small vessel disease (CSVD). EPVS are common among the healthy elderly population. While previous studies focused on EPVS among midlife and older adults over 55 years of age, no prior literature investigated the clinical significance of EPVS in young adults from a neurology clinic-based cohort (≤45 years) [1,2]. Perivascular spaces (PVS), also known as Virchow–Robin spaces, are the interstitial fluid-filled areas surrounding the walls of small cerebral arteries, capillaries, and small veins [3]. The cerebrospinal fluid (CSF) maintains the balance of the intracerebral environment by clearing metabolic waste through the PVS, which in turn flows into the brain parenchyma. Current research suggests that β-amyloid, tau protein deposition, and decreased expression levels of aquaporin-4 (AQP4) channel proteins are closely associated with reduced function of the PVS [4,5,6]. PVS cannot be observed on conventional MRI but can be visualized when dilated.

The EPVS are linked to the occurrence and severity of CSVD, serving as one of the characteristic signs of the disease [7,8,9]. Extensive research shows that EPVS increase in number and volume with age [10]. The EPVS are also reported to be associated with cognitive impairment [11], multiple sclerosis [12], Parkinson’s disease [13], Alzheimer disease [14], internal carotid artery stenosis [15], and intracerebral hemorrhage [16].

In our clinical practice, we frequently observed EPVS without specific cause among young adult patients. Since EPVS are primarily studied in the older population, the significance of EPVS among young patients is relatively undocumented. The purpose of the present study was to investigate the prevalence and risk factors of EPVS among young adults who have no specific cause from a neurology clinic-based cohort (≤45 years old) to improve our understanding of EPVS in this population.

## 2. Materials and Methods

### 2.1. Study Population

**Figure 1:**
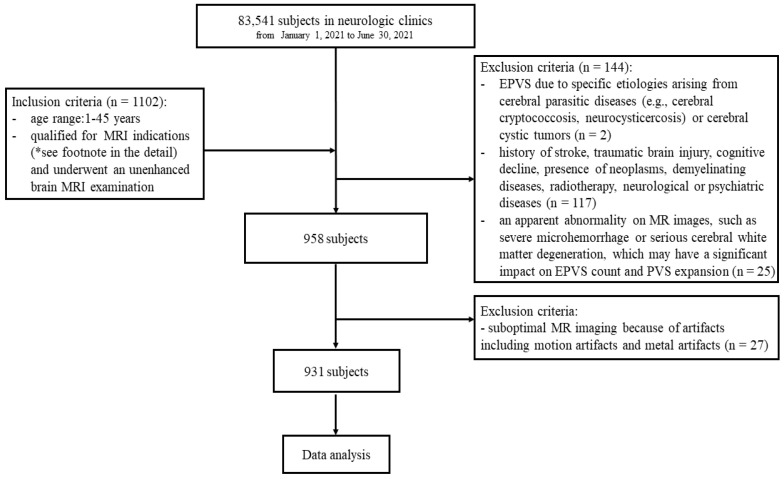
Subject screening workflow. *, MRI indications were defined as follows: (1) an increase in the frequency and severity of symptoms, such as headache, which may indicate secondary pathology (*n* = 797); (2) any abnormal neurologic finding, including asymmetric or unilateral hearing or vision loss (*n* = 238); and (3) no neurologic symptoms, with head examinations as part of physical examination (*n* = 67).

First, we screened all subjects (*n* = 83,541) who were treated in the neurologic clinics at Shanghai Jiao Tong University Affiliated Sixth People’s Hospital between 1 January 2021 and 30 June 2021. Among this population, we searched for individuals under 45 years old. We included subjects (*n* = 1102) who presented with our MRI indications and underwent an unenhanced brain MRI examination. The MRI indications were defined as follows: (1) an increase in the frequency and severity of symptoms, such as headache, that may indicate secondary pathology (*n* = 797); (2) any abnormal neurologic finding, including asymmetric or unilateral hearing or vision loss (*n* = 238); and (3) no neurologic symptoms with brain examinations as part of the physical examination (*n* = 67). We excluded subjects (*n* = 144) according to the following criteria: (1) EPVS due to specific etiologies arising from cerebral parasitic diseases (e.g., cerebral cryptococcosis and neurocysticercosis) or cerebral cystic tumors (*n* = 2); (2) history of stroke, traumatic brain injury, cognitive decline, presence of neoplasms, demyelinating diseases, radiotherapy, and neurological or psychiatric diseases (*n* = 117); or (3) apparent abnormality in MR images, such as severe microhemorrhage or serious cerebral white matter degeneration, which may have a significant impact on EPVS count and PVS expansion (*n* = 25). A total of 958 subjects met all the above criteria. Since the 27 subjects had suboptimal MR imaging because of artifacts, including motion artifacts and metal artifacts, we included 931 individuals in our study (Figure 1).

This study was approved by the local institutional review board. Due to the retrospective nature of the study, we obtained a waiver for informed consent.

### 2.2. Definition of Symptoms

Symptoms were identified based on the patient’s chief complaint. For some of the symptoms that were more difficult to identify, the following definitions were used.

Dizziness: A sensation of swaying and instability during movement or vision, most commonly while walking, sitting, or lying down.

Vertigo: A sensation (motion hallucination) characterized by episodes of an objectively non-existent but subjective belief that oneself and/or external objects rotate or roll in a specific direction.

Light-headedness: A persistent feeling of light-headedness or mental disorientation.

Somatic symptoms: Multiple recurrent and frequently changing somatic symptoms that can affect any part of the body or organs, but with no obvious organic lesions found through various medical examinations.

Sleep disturbance: A subjective experience of inadequate sleep duration and/or quality that interferes with social functioning during the day. This may include (1) prolonged sleep latency (sleep onset taking more than 30 minutes); (2) sleep maintenance disorder (two or more awakenings during the night or early awakenings in the morning); (3) decreased sleep quality (light sleep and excessive dreaming); (4) shortened total sleep time (usually less than six hours); or (5) residual daytime effects (dizziness, mental fatigue, drowsiness, fatigue, and so on the next morning).

Most of the patients (92.9% [865/931]) experienced clinical symptoms, including 147 patients with unspecified symptoms whose MRI requisition forms mentioned "suspected cerebrovascular disease.” The 66 patients (7.1%) with no neurologic symptoms underwent brain examinations as part of physical examination.

### 2.3. MRI Acquisition

All subjects were scanned using the uniform 3.0T MRI scanning protocol (Magnetom Verio, Siemens Healthcare, Germany). The MRI scanning protocol included axial T1-weighted, axial T2-weighted, axial FLAIR, axial DWI, and sagittal T1-weighted sequences.

(1) T1-weighted: TR = 2000 ms, TE = 9 ms, flip angle = 150°, matrix = 384 × 384 pixels, FOV = 250 mm × 250 mm, slice thickness = 6 mm.

(2) T2-weighted: TR = 6000 ms, TE = 9 ms, flip angle = 150°, matrix = 384 × 384 pixels, FOV = 250 mm × 250 mm, slice thickness = 6 mm.

(3) FLAIR: TR = 8500 ms, TE = 94 ms, flip angle = 150°, matrix = 256 × 180 pixels, FOV = 230 mm × 220 mm, slice thickness = 6 mm.

(4) DWI: TR = 5400 ms, TE = 94 ms, flip angle = 150°, matrix = 162 × 162 pixels, FOV = 229 mm × 229 mm, b-values = 0, 1000 s/mm^2^, slice thickness = 6 mm, intersection gap = 1.8 mm.

### 2.4. Data Collection

As part of this study, we collected information on patient demographics (age and gender), clinical symptoms, and vascular risk factors (hypertension, diabetes, and hyperlipemia).

The MRI results were evaluated by an experienced neuroradiologist (Q.Q.Z.) blinded to the clinical data. Cohen kappa statistics were used to determine the interrater reliability for EPVS, as evaluated on a random sample of 100 subjects with a four-week interval between the first and second image assessments. EPVS were characterized by low signal intensities on T1-weighted and FLAIR images and a high signal intensity on T2-weighted images, which was consistent with the alignment of the penetrating vessels (round, oval, or long). Based on previous findings, we counted the visible PVS in the CSO, BG, and MB [17]. The PVS rating scores used in this study were defined as follows. For the centrum semiovale, the level with the highest amount of CSO-EPVS was selected and graded according to the following criteria: (1) 0: no EPVS; (2) 1: ≤10 EPVS; (3) 2: 11–20 EPVS; (4) 3: 21–40 EPVS; and (5) 4: >40 EPVS [17]. When the number was not bilaterally consistent, the side with the higher number of EPVS was used for analysis. For the basal ganglia, the level with the highest number of BG-EPVS was selected and graded as follows: (1) 1: <5 EPVS; (2) 2: 5–10 EPVS; (3) 3: 10–20 EPVS; and (4) 4: >20 EPVS [17]. The midbrain was graded as follows: 0 for no visible EPVS and 1 for visible EPVS [18].

### 2.5. Statistical Analysis

Statistical tests were performed using SPSS for Microsoft Windows software (version 26.0, IBM, Armonk, NY, USA). We dichotomized PVS scores for each brain region according to the median value [19]. The CSO-EPVS degree was dichotomized into low (score 0–1) and high (score 2–4) based on its median value of 1. The BG-EPVS score was dichotomized into low (score 1) and high (score 2–4) based on its median value of 1. Categorical variables were summarized as numbers and percentages. Each symptom was treated as an individual binary variable. Univariate analysis was performed using chi-square testing, and the variables with *p* < 0.2 were included in our logistic regression analysis. Dichotomous logistic regression analyses were applied to determine the risk factors of EPVS (*p* < 0.05). Kendall’s rank correlation was used to evaluate the associations between EPVS in each brain region, and *p* < 0.05 was considered statistically significant.

## 3. Results

### 3.1. Baseline Features

Table 1 summarizes the demographic, clinical, and imaging data of the cohort (*n* = 931; male = 446 (47.9%), female = 485 (52.1%); median age: 34 years (28, 40 years); age range: 7–45 years). A total of 37 (4.0%) of the 931 subjects presented with diabetes mellitus, 84 (9.0%) had hypertension, and 32 (3.4%) had hyperlipemia. Most of the individuals experienced symptoms (92.9%), where 26.2% (244/931) had headache, 18.4% (171/931) had dizziness, 3.9% (36/931) had vertigo, 2.0% (19/931) had syncope, 3.2% (30/931) had light-headedness, 7.4% (69/931) had somatic symptoms, 3.4% (32/931) had hearing disturbances, 2.1% (20/931) had visual disturbances, 5.3% (49/931) had convulsions, 2.3% (21/931) had tremors, 2.9% (27/931) had sleeping disturbances, and 15.8% (147/931) had unspecified symptoms. The overall prevalence of EPVS in our study was 99.8% (929/931). CSO-EPVS were observed in 98.0% (910/929) of the individuals with EPVS, while BG-EPVS were observed in 90.6% (842/929) and MB-EPVS in 58.4% (543/929). The CSO-EPVS, BG-EPVS, and MB-EPVS were predominantly scored as 1 (52.1%, 79.1%, and 58.3%, respectively).

### 3.2. Chi-Square Testing

As shown in Table 2 and Table 3, chi-square testing indicated that age (*p* < 0.001), hypertension (*p* = 0.001), and dizziness (*p* = 0.036) were significantly associated with the degree of CSO-EPVS. Furthermore, age (*p* < 0.001), diabetes (*p* = 0.003), hypertension (*p* < 0.001), and dizziness (*p* = 0.042) were significantly associated with the degree of BG-EPVS. Diabetes (*p* = 0.004), hypertension (*p* < 0.001), headache (*p* = 0.005), dizziness (*p* = 0.023), and vertigo (*p* = 0.039) were associated with the degree of MB-EPVS. Sleep disturbance was not associated with CSO-EPVS (*p* = 0.246), BG-EPVS (*p* = 0.195), or MB-EPVS (*p* = 0.276).

### 3.3. Logistic Regression Analysis

Table 4 presents the results of logistic regression analysis for the predictors of severe CSO-EPVS, BG-EPVS, and MB-EPVS. The 31- to 45-year-old age group (OR = 2.544, *p* = 0.004) and hypertension (OR = 1.824, *p* = 0.014) were associated with CSO-EPVS degree. The 16- to 30-year-old age group was not associated with CSO-EPVS (*p* = 0.053). Increasing age (16–30: OR = 8.466, *p* = 0.037; 31–45: OR = 17.021, *p* = 0.005) and hypertension (OR = 2.766, *p* < 0.001) were associated with BG-EPVS degree. Diabetes (OR = 2.938, *p* = 0.014), hypertension (OR = 4.317, *p* < 0.001), headache (OR = 1.807, *p* = 0.001), and dizziness (OR = 1.574, *p* = 0.025) were associated with the degree of MB-EPVS.

### 3.4. Kendall’s Rank Correlation

We observed a positive correlation between BG-EPVS and either CSO-EPVS or MB-EPVS (τ-b = 0.221, *p* < 0.001; τ-b = 0.139, *p* < 0.001), respectively. There was also a positive correlation between CSO-EPVS and MB-EPVS (τ-b = 0.069, *p* = 0.036).

## 4. Discussion

In the present study, we investigated the prevalence and the risk factors of EPVS without specific cause in different brain areas among young adults from a neurology clinic-based cohort. Previous studies identified an association between age and CSO-EPVS, but no significant correlations involving gender, hypertension, diabetes, or hyperlipidemia [20]. The main risk factors for BG-EPVS are age and hypertension, and there are no significant correlations for gender, diabetes, or hyperlipidemia [20]. While few studies were conducted on the risk factors of MB-EPVS, the available research suggests no correlations between MB-EPVS and age, gender, hypertension, or hyperlipidemia [18]. However, it is worth noting that there is a lack of research on the association between diabetes and MB-EPVS. A large number of studies were conducted on EPVS, but its pathophysiology remains unclear [21]. The two most common pathogenic mechanisms are as follows. In the first mechanism, an intrinsic process affects the deep small penetrating arteries (BG, deep white matter, and MB), which may involve small arteriosclerosis, lipohyalinosis, or fibrinoid necrosis, but is usually thought to be related to hypertension. The second mechanism is mainly attributed to cerebral amyloid angiopathy (CAA). While the former mechanism preferentially occurs in the deep penetrating arteries (BG and MB), CAA usually affects the cortical and soft meningeal vessels [22]. This may be a result of the different anatomies of EPVS within different brain regions [8].

In this study, the overall prevalence of EPVS was 99.8%. Most subjects had EPVS scores of 1 in all brain regions. Surprisingly, we did not find conclusive evidence in our cohort that certain expected factors were associated with EPVS, although we did confirm most of the expected risk factor associations. More specifically, age, hypertension, diabetes, headache, and dizziness may be related to the severity of EPVS in the corresponding brain areas.

Our study also evaluated the severity of EPVS within different brain regions among young adults from our neurology clinic-based cohort. We predominantly observed CSO-EPVS scores of 1. This differed from the results of Yusuke et al., who mostly saw scores of 2 (51.2%) [19]. The lower CSO-EPVS scores in our study may be related to the fact that our participants were younger than those in the study from Yusuke et al. We mainly observed BG-EPVS scores of 1, which was consistent with the results of Shuna et al. [17]. This was not surprising—although the participants in our study were quite young, most were symptomatic. Furthermore, the 31- to 45-year-old age groups experienced higher degrees of CSO-EPVS compared to the 1- to 15-year-old group, but the 16- to 30-year-old age group did not. This was inconsistent with previous studies, but it is possible that the vast majority of subjects in our cohort had symptoms. In addition, the 16- to 30-year-old and 31- to 45-year-old age groups experienced higher degrees of BG-EPVS compared to the 1- to 15-year-old group. In line with previous reports, the degree of EPVS increased with the age groups [6,23,24,25].

We found that hypertensive patients exhibited more profound EPVS in the BG. Due to the vertical alignment of the penetrating arteries in the BG, these structures are more susceptible to elevated blood pressure. The blood–brain barrier (BBB) is more vulnerable to disruption as a result of high blood pressure, which increases the likelihood of impaired lymphatic system clearance and leads to EPVS [3]. In addition, the data show similar results in the CSO and MB for hypertensive patients. Although previous studies failed to identify a direct association between hypertension and either CSO-EPVS or MB-EPVS [4,5], some studies discovered a link between stroke and cerebral small vessel disease [26]. There are a large number of studies on the pathogenesis between stroke and hypertension. Hypertension causes damage to vascular endothelial cells, severe inflammation, and a hypercoagulable state of blood, all of which disrupt the BBB [26,27]. Furthermore, irrational antihypertensive drug selection contributes to the occurrence of stroke [28]. As a result, we could deduce that the aforementioned pathogenesis may also be the primary reason why hypertension was associated with the severity of EPVS. This result led to our conclusion that hypertensive patients demonstrate more severe CSO-EPVS and MB-EPVS. Therefore, in clinical practice, radiologists working with young hypertensive patients should not only pay attention to changes in the BG, but also to those in the CSO and MB.

Many studies found that diabetes mellitus is not a significant predictor for EPVS. However, our study identified an association between diabetes mellitus and MB-EPVS. Previous research suggests that hyperglycemia can lead to cerebral microvascular dysfunction, pathological neurovascular remodeling, BBB disruption, and neuron and glial cell damage [29,30]. Moreover, Sink et al. demonstrated that diabetic patients are prone to develop white matter lesions and brain atrophy [31]. In prior studies, many researchers noted a correlation between white matter lesions and EPVS, suggesting their potential application as biomarkers for CSVD. Consequently, this may indicate a potential relationship with increased MB-EPVS. However, due to the small sample size and low prevalence of diabetes (4% [37/931]) among the outpatients in the present study, a larger sample size is required to confirm our conclusions.

Previous research demonstrated that tau and Aβ can be removed during sleep [32,33]. Therefore, changes in the sleep–wake cycle can lead to the deposition of proteins and other products that cause perivascular spaces to enlarge in certain regions of the brain. Lysen et al. found that poor sleep is associated with a higher CSO-EPVS load among older adults [34]. Del et al. showed that healthy subjects >60 years of age with sleep deficiencies tend to develop BG-EPVS [35]. In contrast to previous research, we did not observe an association between sleep disturbance and EPVS in any of the considered brain regions. We postulate that the main reason for this discordant result is the younger age of the subjects in our study, who collectively experienced a shorter duration of less severe symptoms.

To the best of our knowledge, no previous studies evaluated the relationship between neurological symptoms and the severity of EPVS. Our study indicated that neurological symptoms significantly impacted MB-EPVS. More specifically, we found that patients with headache and dizziness were more likely to develop MB-EPVS compared to those without neurological symptoms. Therefore, radiologists should pay closer attention to the patient’s clinical history and perform detailed assessments of accompanying symptoms.

The present study exhibits certain limitations. First, our study did not include information on confounding factors, such as physical exercise [36], smoking [19], drug treatment, and obesity [37]. The results of previous studies suggest that obesity, and especially abundant visceral adipose tissue, plays a role in the formation of EPVS. Second, we did not quantitatively assess EPVS. Further research is warranted to investigate the underlying causes of EPVS in young adults. The computationally derived PVS metrics (total volume and count, individual size, length, and width) should be used for a more precise evaluation of EPVS [38]. Third, our sample was potentially subject to selection bias, considering the indications for brain MRI vary from institution to institution. Finally, our study had a relatively small sample size and was limited by its retrospective nature. In the future, a multicenter, larger sample with a control group study should be conducted to confirm our findings.

## 5. Conclusions

In this retrospective study, EPVS findings in MRIs of the head were reinterpreted in a group of young people under 45 years of age from a neurology clinic-based cohort. EPVS were frequently found in young adults and could be related to the symptoms. Age, hypertension, and diabetes were the risk factors for the severity of EPVS in the corresponding brain regions. In future clinical practice, it would be beneficial to pay attention to EPVS in young outpatients in MR imaging for clinical management of examiners.

## Figures and Tables

**Table 1:** Basic data on patient demographics, clinical symptoms, and imaging manifestations.

		0–15 years (*n* = 58)	16–30 years (*n* = 258)	31–45 years (*n* = 615)
Gender	Male, *n* (%)	29 (50.0%)	119 (46.1%)	298 (48.5%)
	Female, *n* (%)	29 (50.0%)	139 (53.9%)	317 (51.5%)
Vascular risk factors	Diabetes, *n* (%)	0 (0.0%)	3 (1.2%)	34 (5.5%)
	Hypertension, *n* (%)	1 (1.7%)	11 (4.3%)	72 (11.7%)
	Hyperlipidemia, *n* (%)	0 (0.0%)	1 (0.4%)	31 (5.0%)
Symptoms	Absent, *n* (%)	13 (22.4%)	14 (5.4%)	39 (6.3%)
	Headache, *n* (%)	16 (27.6%)	81 (31.4%)	147 (23.9%)
	Dizziness, *n* (%)	4 (6.9%)	43 (16.7%)	124 (20.2%)
	Vertigo, *n* (%)	0 (0.0%)	12 (4.7%)	24 (3.9%)
	Syncope, *n* (%)	2 (3.4%)	9 (3.5%)	8 (1.3%)
	Light-headedness, *n* (%)	1 (1.7%)	6 (2.3%)	23 (3.7%)
	Somatic symptoms, *n* (%)	0 (0.0%)	16 (6.2%)	53 (8.6%)
	Hearing disturbances, *n* (%)	2 (3.4%)	7 (2.7%)	23 (3.7%)
	Visual disturbances, *n* (%)	1 (1.7%)	5 (1.9%)	14 (2.3%)
	Convulsions, *n* (%)	1 (1.7%)	18 (7.0%)	30 (4.9%)
	Tremors, *n* (%)	0 (0.0%)	8 (3.1%)	13 (2.1%)
	Sleeping disturbances, *n* (%)	1 (1.7%)	8 (3.1%)	18 (2.9%)
	Unspecified, *n* (%)	17 (29.3%)	31 (12.0%)	99 (16.1%)
Prevalence	CSO-EPVS, *n* (%)	43 (74.1%)	255 (98.8%)	614 (99.8%)
	BG-EPVS, *n* (%)	39 (67.2%)	224 (86.8%)	579 (94.1%)
	MB-EPVS, *n* (%)	38 (65.5%)	135 (52.3%)	370 (60.2%)
CSO-EPVS score	0, *n* (%)	15 (25.9%)	3 (1.2%)	1 (0.2%)
	1, *n* (%)	29 (50.0%)	150 (58.1%)	306 (49.8%)
	2, *n* (%)	13 (22.4%)	90 (34.9%)	255 (41.5%)
	3, *n* (%)	1 (1.7%)	12 (4.7%)	49 (8.0%)
	4, *n* (%)	0 (0.0%)	0 (0.0%)	4 (0.7%)
BG-EPVS score	1, *n* (%)	57 (98.3%)	223 (86.4%)	456 (74.1%)
	2, *n* (%)	1 (1.7%)	34 (13.2%)	147 (23.9%)
	3, *n* (%)	0 (0.0%)	0 (0.0%)	7 (1.1%)
	4, *n* (%)	0 (0.0%)	1 (0.4%)	5 (0.8%)
MB-EPVS score	0, *n* (%)	20 (34.5%)	123 (47.7%)	245 (39.8%)
	1, *n* (%)	38 (65.5%)	135 (52.3%)	370 (60.2%)

**Table 2:** The results of the chi-square analysis of the differences in the demographics, vascular factors, and MRI findings between the groups according to the degree of EPVS.

	CSO-EPVS		*p*	BG-EPVS		*p*	MB-EPVS		*p*
	Low: 0–1 (*n* = 519)	High: 2–4 (*n* = 412)		Low: 1 (*n* = 734)	High: 2-4 (*n* = 197)		Low: 0 (*n* = 388)	High: 1 (*n* = 543)	
Age			<0.001			<0.001			0.052
1–15 y, *n* (%)	44 (8.5) [3.2]	14 (3.4) [−3.2]		57 (7.8) [3.7]	1 (0.5) [−3.7]		20 (5.2) [−1.1]	38 (7.0) [1.1]	
16–30 y, *n* (%)	156 (30.1) [1.8]	102 (24.8) [−1.8]		223 (30.4) [3.5]	35 (17.8) [−3.5]		123 (31.7) [2.3]	135 (24.9) [−2.3]	
31–45 y, *n* (%)	319 (61.5) [−3.3]	296 (71.8) [3.3]		454 (61.9) [−5.2]	161 (81.7) [5.2]		245 (63.1) [−1.6]	370 (68.1) [1.6]	
Male, *n* (%)	235 (45.3)	211 (51.2)	0.072	342 (46.6)	104 (52.8)	0.122	180 (46.4)	266 (49.0)	0.434
Diabetes, *n* (%)	16 (3.1)	21 (5.1)	0.118	22 (3.0)	15 (7.6)	0.003	7 (1.8)	30 (5.5)	0.004
Hypertension, *n* (%)	32 (6.2)	52 (12.6)	0.001	46 (6.3)	38 (19.3)	<0.001	13 (3.4)	71 (13.1)	<0.001
Hyperlipidemia, *n* (%)	13 (2.5)	19 (4.6)	0.080	21 (2.9)	11 (5.6)	0.063	10 (2.6)	22 (4.1)	0.223

Note: [corrected residual].

**Table 3:** The results of the chi-square analysis of the differences in the clinical symptoms and MRI findings between the groups according to the degree of EPVS.

	CSO-EPVS		*p*	BG-EPVS		*p*	MB-EPVS		*p*
	Low: 0–1 (*n* = 519)	High: 2–4 (*n* = 412)		Low: 1 (*n* = 734)	High: 2–4 (*n* = 197)		Low: 0 (*n* = 388)	High: 1 (*n* = 543)	
Absent, *n* (%)	36 (6.9)	30 (7.3)	0.839	53 (7.2)	13 (6.6)	0.763	32 (8.2)	34 (6.3)	0.244
Headache, *n* (%)	128 (24.7)	116 (28.2)	0.229	195 (26.6)	49 (24.9)	0.631	83 (21.4)	161 (29.7)	0.005
Dizziness, *n* (%)	83 (16.0)	88 (21.4)	0.036	125 (17.0)	46 (23.4)	0.042	58 (14.9)	113 (20.8)	0.023
Vertigo, *n* (%)	22 (4.2)	14 (3.4)	0.509	30 (4.1)	6 (3.0)	0.501	21 (5.4)	15 (2.8)	0.039
Syncope, *n* (%)	11 (2.1)	8 (1.9)	0.849	16 (2.2)	3 (1.5)	0.563	7 (1.8)	12 (2.2)	0.666
Light-headedness, *n* (%)	15 (2.9)	15 (3.6)	0.519	21 (2.9)	9 (4.6)	0.228	11 (2.8)	19 (3.5)	0.572
Somatic symptoms, *n* (%)	40 (7.7)	29 (7.0)	0.699	51 (6.9)	18 (9.1)	0.298	35 (9.0)	34 (6.3)	0.113
Hearing disturbance, *n* (%)	20 (3.9)	12 (2.9)	0.434	25 (3.4)	7 (3.6)	0.920	18 (4.6)	14 (2.6)	0.089
Visual disturbance, *n* (%)	12 (2.3)	8 (1.9)	0.699	18 (2.5)	2 (1.0)	0.217	10 (2.6)	10 (1.8)	0.445
Convulsion, *n* (%)	29 (5.6)	20 (4.9)	0.619	41 (5.6)	8 (4.1)	0.395	25 (6.4)	24 (4.4)	0.173
Tremor, *n* (%)	14 (2.7)	7 (1.7)	0.308	17 (2.3)	4 (2.0)	0.811	9 (2.3)	12 (2.2)	0.912
Sleep disturbance, *n* (%)	18 (3.5)	9 (2.2)	0.246	24 (3.3)	3 (1.5)	0.195	14 (3.6)	13 (2.4)	0.276
Unspecified, *n* (%)	91 (17.5)	56 (13.6)	0.101	118 (16.1)	29 (14.7)	0.643	62 (16.0)	85 (15.7)	0.893

**Table 4:** Logistic regression analysis for independent predictors of EPVS degree in different regions of the brain.

	OR	95% CI	*p*
CSO-EPVS			
Age			0.005
16–30 years	1.910	0.991–3.682	0.053
31–45 years	2.544	1.356–4.772	0.004
Female	0.857	0.655–1.120	0.258
Diabetes	1.307	0.651–2.624	0.451
Hypertension	1.824	1.128–2.951	0.014
Hyperlipidemia	1.260	0.590–2.691	0.550
Dizziness	1.225	0.865–1.734	0.253
Unspecified	0.778	0.533–1.137	0.195
BG-EPVS			
Age			<0.001
16–30 years	8.466	1.133–63.266	0.037
31–45 years	17.021	2.329–124.382	0.005
Female	0.921	0.659–1.288	0.632
Diabetes	1.829	0.888–3.768	0.101
Hypertension	2.766	1.695–4.514	<0.001
Hyperlipidemia	0.991	0.442–2.223	0.983
Dizziness	1.253	0.840–1.870	0.268
Sleep disturbance	0.505	0.147–1.729	0.276
MB-EPVS			
Age			0.087
16–30 years	0.533	0.290–0.982	0.043
31–45 years	0.676	0.378–1.210	0.187
Diabetes	2.938	1.214–6.956	0.014
Hypertension	4.137	2.324–8.018	<0.001
Headache	1.807	1.272–2.556	0.001
Dizziness	1,574	1.059–2.340	0.025
Vertigo	0.667	0.327–1.360	0.265
Somatic symptoms	0.727	0.421–1.258	0.255
Hearing disturbance	0.632	0.298–1.341	0.232
Convulsion	0.883	0.478–1.631	0.691

Note: boldface type indicates statistical significance. The variables with *p* < 0.2 in the univariate analysis were included in the logistic regression analysis.

## Data Availability

The data supporting the findings of this study are available from the corresponding author upon reasonable request.

## Abbreviations

EPVS	enlarged perivascular spaces;
CSO	centrum semiovale;
BG	basal ganglia;
MB	midbrain;
PVS	perivascular spaces;
CSF	cerebrospinal fluid;
CSVD	cerebral small vessel disease;
AQP4	aquaporin-4;
CAA	cerebral amyloid angiopathy;
BBB	blood–brain barrier;
OR	odds ratio;
CI	confidence interval.
